# Development of the BELANA questionnaire for the analysis of economic burdens of food allergy and intolerance 

**DOI:** 10.5414/ALX01322E

**Published:** 2018-09-01

**Authors:** N. Rösch, S. Schnadt, R. Herbst, A. Arens-Volland, S. Kohler, F. Feidert, P. Schmalz, K. Hermann, R. Mösges

**Affiliations:** 1 Centre de Recherche Public Henri Tudor, Luxembourg,; 2 Deutscher Allergie- und Asthmabund e.V. (DAAB), Mönchengladbach,; 3 Clinique d’Eich, Centre Hospitalier de Luxembourg,; 4 Institut für Medizinische Statistik, Informatik und Epidemiologie der Universität zu Köln (IMSIE)

**Keywords:** food allergy, food intolerance, health economic evaluation, health-related quality of life, item development, patient orientation, web based survey, pilot trial, WikiFood.eu, SF-36, SF-12, FAQLQ-AF

## Abstract

Introduction: Patients affected by food allergies and intolerance need to apply dedicated avoidance strategies and also prevent the consequences of unbalanced diets. In most countries, the health economic costs for these patients are unknown. Methods: To measure temporal and financial burdens of the patients in multinational settings, the BELANA questionnaire (Burdens and Expenses of Living as an Adult with Nutrition based Allergy or Intolerance) has been developed. For the complementary measurement of Health Related Quality of Life (HR-QoL), a combined appliance of the disease-specific FAQLQ-AF (Food Allergy Quality of Life Questionnaire – Adult Form) and the generic SF-12v1 (Short Form-12 Health Survey) has been chosen. Results: BELANA collects six economic items while avoiding questions, which are already included in the HR-QoL questionnaires or could lead to denial tendencies. In a web-based pilot survey with 51 patients, the practicability of using BELANA together with the complementary quality of life instruments was investigated. The electronic data collection offers real time plausibility checks and limits the workload for completion and data evaluation. Discussion: The response rates at BELANA health-economic items (76 – 100%) and the high amount of completed questionnaires (50 of 51) confirm the patients acceptance of the chosen methodology. Within the web-based survey, the combination of BELANA, FAQLQ-AF and SF-12v1 was completed in an average of 22 minutes. An age-related selection bias was not been confirmed in this pilot application. The median age in the pilot trial was 37.9 years (minimum age to participate was 18 years, range from 19 to 72 years, Standard Deviation (SD) = 12.4 years). Most of the participants were female (44 of 50). Conclusion: It is assumed that the BELANA questionnaire should be a useful tool for the evaluation of health-economic burden for patients with food allergy and intolerance.

**German version published in Allergologie, Vol. 34, No. 4/2011, p. 177-190**

## Introduction 

Due to the high number of affected persons and the broad public interest food allergies and intolerance have to be considered as health-related problems with increasing public health relevance [21, 30]. To date, no causal short-term therapies for long-term cure are available for these chronic diseases. For some food allergies (e.g., to hazelnut, egg, milk) specific sublingual immunotherapy (SLIT) or specific oral tolerance induction (SOTI) are possible in principle, but are predominantly restricted to clinical trials where a close monitoring, according to the risks, is possible [8, 17, 38]. If a pollen-associated food allergy is accompanied by pollen-dependent respiratory complaints, subcutaneous specific immunotherapy (SCIT) might be an option [17, 22]. 

The focus of medical treatment has to be on individual dietary management in order to avoid contact with the symptom-triggering food components. The fact that patients have to avoid certain foods permanently and that they have to be extremely careful with the choice and preparation of foods leads to a marked impairment of their everyday lives. Pharmaceutical products are mainly supposed to palliate the symptoms or to treat diet mistakes. Thus, patients have to learn comprehensive avoidance strategies and apply them permanently but at the same time maintain a well-balanced nutrition. In addition, the resulting impairment of the patients’ quality of life can be accompanied by significant expenses [32]. 

The FAQLQ-AF (Food Allergy Quality of Life Questionnaire – Adult Form), recently presented by Flokstra-de Blok et al. [12], is a disease-specific instrument for the measurement of the health-related quality of life (HR-QoL) of patients with food allergies. The reduction in quality of life is, however, only a part of the total burden of the patients. So far, there are not sufficient studies allowing for the quantification of impairments in everyday life caused by food allergies and intolerance. The existing facts on sickness and absence from work are mostly based on working patients [32]. 

We here present for the first time the BELANA questionnaire (Burdens and Expenses of Living as an Adult with Nutrition based Allergy or Intolerance) that contains 10 questions to evaluate 6 predefined economic parameters from the patient’s point of view. The BELANA questionnaire has been developed as a part of the international patient-oriented MENSSANA project by the Luxembourg Research Center “CRP Henri Tudor”, “Centre Hospitalier de Luxembourg“, “Deutscher Allergie- und Asthmabund e.V. (DAAB)”, and “Institut für Medizinische Statistik, Informatik und Epidemiologie der Universität zu Köln (IMSIE)”. 

## Methods 

### Choice of study perspective 

To analyze the health-economic burden of a disease it is always necessary to choose an adequate study perspective [10]. A whole society perspective would show whether certain measures lead to a redistribution of the burden to other cost units (e.g., the patient’s). However, the efforts necessary for such an approach can only be accomplished in large-scale trials [36]. Another possible perspective, the health payers point of view, would only take into account those cost types that burden their budgets [20] making all costs born by the patients, their relatives and employers irrelevant. 

More and more healthcare costs are passed on to the patients. In Germany, various co-payments, like quarterly flat-rate charges for ambulatory treatment, or increased co-payments for drugs, as well as the reduction of coverage lead to a significant economic burden, particularly for the chronically ill [13]. Trips to see the doctor, going to the pharmacy or preparing special meals cost additional time and money. Thus, it makes sense to investigate the economic consequences of food allergies and intolerance primarily from the patient’s perspective. In order to assess not only the quantifiable economic burden but also the intangible costs, a complementary measurement of the health-related quality of life will be useful [15]. 

### Determination of the target group 

This observational study aims at assessing and analyzing the individual burden and expenses of patients with food allergy or food intolerance. According to the nomenclature of the EAACI (European Academy of Allergy and Clinical Immunology) and the WAO (World Allergy Organization) immunologically mediated food sensitivities are termed “allergies”. The term “intolerance” is used for all non-immunologically mediated food sensitivities. Among these are reactions of unknown etiology (e.g., intolerance to food additives) as well as enzymopathies like lactose intolerance [17]. In order not to confuse the patients immunologically mediated gluten sensitivity (celiac disease) was – as suggested by Jäger et al. [17] – counted among the intolerance in the questionnaire. Regardless of this, it is still justified to discuss whether celiac disease should be classified as T-cell-mediated food allergy [37]. 

For clinicians it is frequently very difficult and time-consuming to clearly identify the trigger of a reaction and they strongly depend on the patient’s cooperation [29]. Real immediate-type food allergies are diagnosed on the basis of patient history, skin tests and oral provocation as well as on the detection of IgE antibodies. For non-immunologically mediated sensitivity reactions there is usually a lack of measurable laboratory parameters [31]. In these cases the presumed triggers need to be applied in complex provocation tests in order to unambiguously reproduce the symptoms. Also patient diaries can be useful to help identify food allergies and intolerance, but precise conclusions are frequently impaired by insufficient notes or unreadable handwriting. At the moment there are promising approaches with electronic diaries [34]. 

### Health scientific dimension of individual burden and expenses 

In Western Europe approximately 30% of the population suffers from allergies. Not only the number of patients but also the intensity of allergic reactions has increased over the past decades [27, 28]. About 15% of the population suffer from hay fever, 10% have allergic asthma and approximately 4% are affected by food allergies [46]. Many patients have to avoid an increasing number of substances and frequently their symptoms worsen [16]. 

Reliable data based on well-designed meta-analyses of the prevalence of specific intolerance reactions to food components are scarce [45]. Vesa et al. [42] report for the USA that approximately 15% of Caucasians and approximately 80% of African Americans suffer from lactose intolerance. For Europe very heterogeneous prevalence rates are reported (2% in Scandinavia, 15% in Germany, 70% in Sicily), while in Asia almost 100% of people are affected [42]. For gluten sensitivity (celiac disease) the data on prevalence varies between 0.1% and 0.4% in Europe, while this disease is very rare in Africans and Chinese [17]. 

While the skin and intestinal symptoms of food allergies and food intolerance are similar, allergic reactions can additionally cause symptoms in other organs [2]. IgE-mediated reactions count among the most frequent elicitors of anaphylaxis, which is the maximum allergic shock reaction and can affect the whole organism [25]. It is assumed that about 8 – 10 of these maximum reactions per 100,000 inhabitants occur each year [7]. A significant number of fatal anaphylactic reactions is caused by accidental ingestion of not tolerated foods (e.g., peanuts or fish). Difficult to read or insufficient labeling of ingredients contributes to uncertainty among patients [5]. Many allergy patients are living with the knowledge that a single diet mistake can lead to a life-threatening situation [4, 24]. To know that one’s life might be threatened causes an emotional burden, particularly in younger people, with hard-to-predict long-term effects [23]. Already non-lifethreatening symptoms like pruritus, urticaria, edema, rhinorrhea, diarrhea, intestinal colic and vomiting result in significant impairments of everyday life. For example, when patients with undiagnosed medical conditions eat in a restaurant or are invited by friends or relatives they cannot define clearly the food they can eat. This unsettles the patient as well as the host. It is considered a fact that the lack of knowledge about possible triggers contributes significantly to the reduced quality of life in patients with food allergies [12]. 

In order to avoid symptoms, patients with allergies and intolerance patients need to know the triggers, and the strategies they have to use are similar. As a consequence it makes sense to develop a measure that can be used to analyze the economic burden of both diseases. Currently, the usefulness of modern information technology (smartphones as mobile diet assistants and web-based information services like www.wikifood.eu) in supporting patients is studied [35]. 

## Results 

### Development of the BELANA questionnaire for the survey of health-economic parameters in food allergy and intolerance 

The BELANA questionnaire was primarily developed to facilitate an international survey of the economic burden of affected patients as well as their expenditure of time. Already at the conception stage we have taken into account the complementary measurement of the health-related quality of life in order to also assess the intangible share of the burden of each patient. The BELANA questionnaire has been designed for paper-based and electronic surveys and was used for the first time in a web-based survey comparing German and Luxembourgian patients within the MENSSANA project (Mobile Expert & Networking System for Systematical Analysis of Nutrition based Allergies) [35]. 

**Financial burden and expenditure of time in food allergy**

The dimension of patients’ co-payments varies within Europe. Despite being health-insured the patients have to raise considerable funds and to spend a significant amount of time for consultations. In Germany quarterly flat-rate charges for ambulatory treatment are applied, increasing the patient’s financial burden. Further financial burden is caused by co-payments for drugs, consultation fees for certain medical services, as well as by transport costs to physicians and pharmacies. Patients using alternative diagnostic or therapeutic methods (e.g., asking the pendulum, IgG4-value, bioresonance, kinesiology, traditional Chinese medicine, homeopathy) do not only have to bear the possible health risks, but also the costs of scientifically disputable procedures [18, 19]. In Germany some health insurances pay for alternative methods in order to achieve an outstanding market position since the premium rate was standardized for all providers in 2009 [39]. 

In some cases even hospitalization becomes necessary which represents another considerable burden. Patients have to leave their familiar surroundings and cannot meet their daily commitments. Hospitalization can become necessary for diagnostic work-up, but also in emergencies resulting from diet mistakes. The health-economic evaluation of drug use requires knowledge about the patient’s total allergy history. Concomitant pollinosis, asthmatic bronchitis and allergic bronchial asthma require drug therapies that can also suppress food-dependent symptoms. In the case of pollen-associated food allergies seasonal changes have to be expected [32]. Another important factor is the time expenditure and financial burden caused by the purchase and preparation of special diet meals. Patients and their families do often not dare to take the risk of buying cheaper, but unkown food, which reduces their possibilites to reduce expenditures [12]. 

**Reduced activities of daily living**

Establishing a diagnosis is essential for successful therapy and thus contributes significantly to the preservation of the patient’s quality of life [12]. When undetermined allergies or intolerance are thought to cause medical conditions, frequently no further diagnostic work-up is made and no therapeutic conclusions are drawn [32]. To which extent food allergies or intolerance lead to reduced performance at home and at work has not been sufficiently documented so far. What is known, is that the frequency of work absence due to food allergies and intolerance is of high economic importance [1]. When patients are unable to work for a longer period, the social security systems usually cannot compensate for the loss of income with possibly extremely negative consequences for a family’s economic situation. It is also difficult to cover such costs of allergies by private insurances. For example, allergic rhinitis during childhood can cause insurance companies to impose higher rates, to refuse to provide occupational disability insurance or to explicitly exclude certain risks [14]. 

Work absence represents only a part of the real strain. From the patient’s perspective also those days have to be taken into account on which they cannot fulfill their commitments at work, at school or at home due to their symptoms. A questionnaire has to assess the number of all sick days of employees, trainees, students and pupils as well as of all non-working people (homemakers, pensioners, unemployed). From the patients’ point of view also sick days on weekends or public holidays should be considered to be a similar strain. 

### Complementary survey of health-related quality of life 

**Generic measurement of health-related quality of life**

To assess the health-related quality of life (HR-QoL) we could resort to the complementary application of existing standardized procedures. The advantage of cross-disease (generic) measuring instruments is that the impact of a certain disease on the quality of life can be compared with other diseases. The advantages and disadvantages of possible measuring instruments have to be evaluated according to study design and type of application, and the adequate instrument has to be chosen [6]. Frequently used instruments are the Sickness Impact Profile (SIP), the Nottingham Health Profile or the Munich Quality of Life Dimensions List (German: Münchner Lebensqualitäts-Dimensionen-Liste – MLDL). However, sometimes certain questionnaires are difficult to use due to a long answering time, missing translations or legal licensing limitations [3]. The most frequently used HR-QoL questionnaire is the Short Form 36 Health Survey (SF-36) with 36 items to assess the construct “quality of life”. The SF-12 is a short version of the SF-36 with only 12 items. The patient’s self-assessment is based on the ascertainment of the mental, physical and social aspects of a chronic disease as well as of the functional aspects of their everyday activities [43]. All SF questionnaires mainly focus on a person’s “performance”. This slightly reduces the spectrum of the assessment, but in the context of food allergies and intolerance this is only of minor importance. Marklund and Ahlstedt [23], for instance, use the SF-36 questionnaire in adolescents in order to investigate the impact of “allergy-like” health conditions on their quality of life [23]. For combined investigation the reduced SF-12 has to be preferred. The reduction to 12 items minimizes the time needed to answer the questions and increases the acceptance by interviewees. The good correlation of the results from both SF variants could be shown in numerous trials [3]. Since modified versions of SF-36 and SF-12 have been presented, the original versions described here are also called SF-36v1 and SF-12v1, respectively. 

An alternative to the SF questionnaires is the EuroQoL (EQ-5D). In contrast to SF-36 the EQ-5D combines all dimensions of HR-QoL to one single index value. The EQ-5D was developed in order to obtain a simple and quick instrument that can be used together with disease-specific questionnaires [15]. The EQ-5D is available in more than 40 languages and can thus be used in pan-European studies. A disadvantage for the evaluation of food allergy and intolerance is the fact that the EQ-5D rather focuses on severe diseases causing pain or long-term care. Questions on problems with washing and dressing or on confinement to bed can confuse patients who are “only” suffering from food allergies or intolerance. Thus, the SF-12 seems to be preferable to the EQ-5D. 

**Disease-specific measurement of quality of life**

Disease-specific instruments are of particular value when the aim is to evaluate the effects of characteristic symptoms and to document changes over time [26]. Food allergies and intolerance are characterized by a particularly wide spectrum of possible symptoms. Within the EuroPrevall project an adequate instrument for patients with food allergies was developed and published as FAQLQ (Food Allergy Quality of Life Questionnaire) [12]. Various variants have been developed in order to meet the demands of the different target groups: children, adolescents, adults and parents of affected children [11, 41]. The validation of the Dutch version of the FAQLQ-AF (Food Allergy Quality of Life Questionnaire – Adult Form) has already been published, and for other languages translations ready for validation are available. For food intolerance the FAQLQ-AF is not explicitly recommended, but as no disease-specific alternatives are available and symptoms as well as avoidance strategies of patients are similar to those of patients with IgE-mediated allergies, the use of the FAQLQ-AF could be interesting from a scientific point of view. In conclusion, there is no alternative to the FAQLQ-AF as a complementary disease-specific measuring instrument for HR-QoL at the moment. The parallel use of a generic instrument is explicitly recommended [12]. 

### Differentiation and item reduction in the BELANA questionnaire 

The first step to reduce the number of items in the BELANA questionnaire would be to avoid questions on facts already covered in the complementary HR-QoL survey. Our study uses the FAQLQ-AF questionnaire validated by B. Flokstra-de Blok et al. [40] that also includes questions of the Food Allergy Independent Measure (FAIM) as well as age, gender and disease status. Furthermore, it collects data on the worst experienced reactions as well as on the types of specialists and healthcare providers so far involved in the diagnostic process [12]. This permits to validate the FAQLQ-AF data used in the BELANA survey with a well-established procedure and to use them for more than one study. 

Based on experience from preliminary investigation BELANA does not contain questions on income, because this could potentially cause the respondents to refuse participation. The loss caused by work absence is a factor that has to be evaluated independently of the patient’s personal income and role in society. Furthermore, the monthly income seems to be irrelevant when taking into account that only an unknown proportion of the income-dependent insurance premium is used for the treatment of the allergy or intolerance. The prices negotiated between physicians and health insurances hardly reflect the real therapy costs. Questions on preferred foods (ranging from certified organic cultivation to convenience food) and traders (retail, mail order, discount stores), that were included in an earlier version of BELANA, only provided difficult-to-evaluate information in a preliminary test. Instead, the current version asks the respondents to estimate their additional costs for food purchase. 

Furthermore, BELANA does not contain questions on the respondent’s health insurance status in order to be independent from the different national health systems. If necessary, patients insured with statutory health insurance in Germany can be recognized by their statements about co-payments for drugs and quarterly flat-rate charges for ambulatory treatment. The differentiation between private and statutory health insurance is a German phenomenon that complicates international comparisons. Also, for German patients the status “statutory health insurance” does not make clear which costs are transferred to the patient, because each health care provider covers other forms of treatment. Some insurers cover the costs of scientifically controversial methods like homeopathy, others refuse such reimbursement [39]. In Germany there are also professions whose healthcare costs are completely paid by the state or Bundesland as well as people who do not have health insurance due to their income situation. Thus, the financial burden caused by food allergy and intolerance should be evaluated without taking into account the state of insurance. 

**Items of the BELANA questionnaire**

The aim of the BELANA questionnaire was to be able to assess the economic burden caused by food allergy in a total of 4 surveys over a period of 12 months. For this purpose 6 health-economic items have been defined:

- costs of medical treatment paid by the patient
- drug use
- extra costs for purchase of special food
- days with reduced activity at home, work and school
- days at which patients had to see a doctor
- days in hospital

Two further questions on additional allergies and diagnostic procedures already carried out make it possible to assess the situation in detail. An additional question inquires into the role of the internet in searching for adequate food. Finding adequate food products requires a high degree of the patient’s own initiative and the healthcare system does not provide sufficient help. Some manufacturers offer useful information on the internet. Also, self-help groups and virtual communities provide target group-specific information on the web [9]. In order to evaluate the acceptance of this medium, it is essential to find out to what extent information provided via the internet is perceived as useful by the patients [44]. The answers given in the questionnaire can for instance be used to evaluate initiatives like the web portal “WikiFood.eu” which was developed in the Luxembourg research project MENSSANA. WikiFood.eu is an independent forum that provides over 13,000 ingredients lists of food and cosmetic products from numerous manufacturers [33]. The 10^th^ question in the BELANA questionnaire aims at the patient’s occupational situation: pupil/student/trainee, employed, unemployed, retired or house wife. 

### Study design of the analysis of burdens and expenses and results of the pilot survey 

For the assessment of the burdens and expenses of food allergy and intolerance an online form has been developed that consists of 66 questions: 10 BELANA questions, 29 questions of the FAQLQ-AF supplemented by 15 questions on patient characteristics and the probability of an allergic reaction (FAIM), and 12 questions of the SF-12v1. The participants for the web-based survey were recruited via Deutscher Allergie- und Asthmabund e.V. (DAAB) using their member lists, the media and web sites. Patients were only included if they confirmed to suffer from food allergy or intolerance. All participants are requested to be available for 4 additional surveys over a period of 1 year. All participants must be of age and have internet access as well as an e-mail address. Furthermore, the participants need to have sufficient command of the German or French language. Study design, data privacy measures and patient information have been verified an approved by the ethics committee of the Medical Department of the University of Cologne. 

From December 2008 to June 2009 we identified 392 patients with food allergy or intolerance who were interested in participating. Already before the survey had started, all possible participants were asked to pay attention to the economic burden. 

The applicability of the BELANA questionnaire was evaluated in a pilot survey in June 2009 in which the time needed to answer the questions, the age structure as well as the answers of 51 randomly chosen patients of the recruitment profile were analyzed. Furthermore, we evaluated the time needed to answer the BELANA questionnaire when the FAQLQ-AF and the SF-12v1 were used complementary. 

## Discussion 

### Electronic data collection 

In general, it must be assumed that mostly people who suffer a lot from their disease are willing to participate in a 12-month observational study. An uncomplicated web form is supposed to facilitate access to the survey and address a broad spectrum of participants. On the other hand a web-based survey could deter elderly participants resulting in age bias. When evaluating the survey, it is essential to taek into account the age distribution of participants [21]. 

The use of a database-based web form made it possible to limit the time needed to answer the questions, to pseudonymize the answers and to evaluate the data. Already when data were entered automated validity checks could be carried out, which would not have been possible in a paper-based survey. Also the risk of manual transmission errors in the statistical evaluation of data (SPSS Statistics 17.0 for MacOSX 10.5.7) can be reduced when data are collected electronically. 

### Characteristics of respondents in the pilot survey 

**Figure 1. Figure1:**
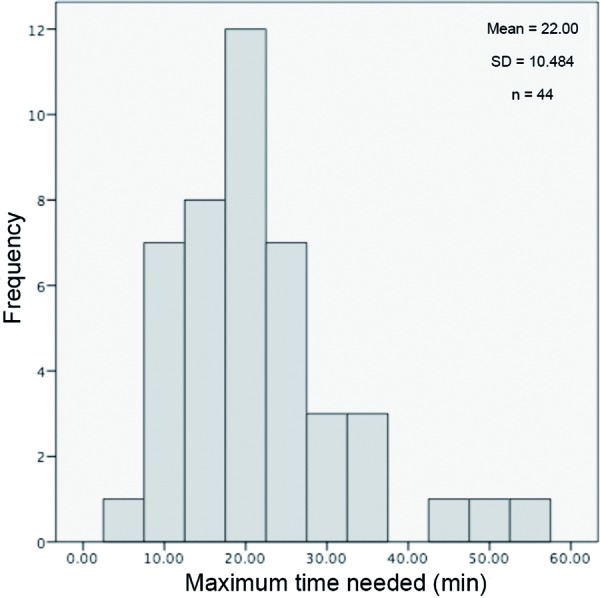
Maximum time needed (in minutes) to answer the questions of the pilot survey.

In a 1-week pilot phase we randomly choose 51 patients with food allergy or intolerance from a total of 392 registered persons. [Fig Figure1]One respondent in the pilot group had not answered all questions. This participant was considered a drop-out and not included in the analysis. The majority of respondents was female (88%) which should be taken into account in gender-specific evaluation. A disproportionate proportion of women has already been observed in other surveys carried out by DAAB. 

**Figure 2.**
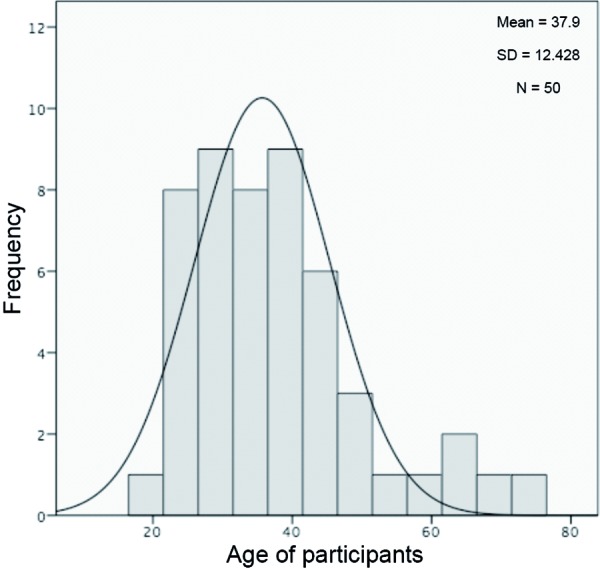
Age distribution of participants of the pilot study.

The age distribution of respondents was analyzed in order to be able to see a possible age-related selection bias (Figure 2). The suspected aversion of elderly people to participate in a web-based survey was not confirmed in the pilot phase. 

The age of the participants was between 19 and 72 years. The mean age was 37.9 years (standard deviation 12.4 years), the minimum age to participate was 18 years. This minimum age could explain the low percentage (8%) of pupils, students and trainees. 62% of respondents were working, 14% were homemakers and 10% had already retired. 

Obviously also the older generation appreciates the general advantages of the internet as a communication medium. In this context it is interesting that only 56% of respondents used the internet to inform themselves about ingredient lists. Services like the online database www.wikifood.eu still need to receive more consideration from the public. 

### Time to answer the questions 

The participants of the pilot study could pause the survey at any time, save their answers and continue later. [Table Table1]The time to answer the 66 questions of BELANA, FAQLQ-AF and SF-12v1 was calculated from the data entry in the system for each participant and is defined as the period of time between the first entry and the completion of the questionnaire. The possible breaks and intermediate savings limit the precision of measurement. If a respondent needed more than 60 minutes to complete the questionnaire it has to be assumed that one or several breaks were made. 44 of 50 participants (90%) could answer all questions in less than 60 minutes (Figure 1). For these 44 respondents the mean time to answer all questions was 22 minutes (standard deviation 10.5 minutes). 

### Response rate for economic items 

In the pilot survey the response rate for the economic items was between 76% and 100%, proving a high level of acceptance (Table 2). 84% of respondents were able to indicate their additional costs for food (item no. 3) and 94% of respondents provided information on their expenses for medical treatment (item no. 1). 66% of respondents had to cover such expenses within the 4 months prior to filling in the questionnaire. Approximately 45% of participants had last seen a doctor more than 4 months before the survey. The preliminary, not yet significant, values from the pilot study suggest estimated mean monthly extra costs of more than EUR 100 for food purchase and more than EUR 20 for medical treatment (Table 3). It has to be further evaluated whether this holds true for larger numbers of participants and whether seasonal differences exist. All participants could answer item no. 6 (days in hospital) and item no. 2 (drug use). As much as 78% of respondents indicated to have needed drugs to alleviate their complaints. Item no. 4 (days with reduced activities of daily living) turned out to be the most difficult question as it was only answered by 76% of respondents. It has to be assumed that the difficulties in answering item no. 4 will particularly occur in the first of the 4 surveys, because its questions are referring to a period of 4 months prior to the start of the survey. Possibly the response rate of certain questions can be increased when the participants can better prepare themselves for those questions. 

## Conclusions 

The analysis of the pilot survey suggest that the BELANA questionnaire is a useful instrument to evaluate the health-economic burden of patients with food allergies and intolerance. 

The web-based form permits to obtain answers from many affected persons within a short period of time and to carry out plausibility checks already while the questionnaire is being answered. An age-related selection bias cannot be completely excluded, but will be manageable when the age distribution is closely observed. To what extent people who suffer a lot from the allergy/intolerance are overrepresented, can only be evaluated after the analysis of burdens and expenses has been completed for a higher number of patients. The complementary survey using BELANA, FAQLQ-AF and SF-12v1 can be answered in an average time of 22 minutes when electronic entry forms are used. Both instruments have been proven to be efficient many times and have not been analyzed again in our pilot study. The patients in the pilot survey had no difficulties in answering the questions of the SF-12v1. In the preliminary studies the participants had complained about the relatively high amount of time needed to answer the FAQLQ-AF, but after conversion to an electronic form there were no further complaints. To what extent FAQLQ-AF can reflect the real quality of life of patients with food intolerance has to be subject to further investigation. 

The analysis of health-economic follow-up costs for chronically ill patients shows the individual and social value of adequate healthcare and can provide important arguments for a goal-oriented healthcare. It is assumed that the BELANA questionnaire will be a useful tool for the evaluation of health-economic burdens for patients with food allergy and intolerance.

**Table 1. Table1:** Characteristics of the respondents in the pilot survey.

	Characteristics of the respondents in the BELANA pilot survey				
	n = 50	Frequency	Percentage	Valid percentage	Accumulated percentage
Age of respondents (from FAQLA-AF)	not specified	0	0.0	0.0	0.0
	> 18 years	50	100.0	100.0	100.0
	> 18 years	0	0.0	0.0	0.0
					
Gender (from FAQLA-AF)	not specified	0	0.0	0.0	0.0
	male	6	12.0	12.0	12.0
	female	44	88.0	88.0	100.0
					
Time needed to answer	< 60 min	45	90.0	90.0	90.0
	> 60 min	5	10.0	10.0	100.0
					
Drug use during the past 4 months	not specified	0	0.0	0.0	0.0
	drugs necessary	39	78.0	78.0	78.0
	no drugs used	11	22.0	22.0	100.0
					
Presence of further allergies	not specified	0	0.0	0.0	0.0
	no	3	6.0	6.0	6.0
	yes	47	94.0	94.0	100.0
					
Diagnostic methods applied (multiple answerspermitted)	not specified	0	0.0	0.0	0.0
	patient history	39	78.0	78.0	–
	skin test (prick etc.)	41	82.0	82.0	–
	IgE determination using RAST	26	52.0	52.0	–
	oral provocation	15	30.0	30.0	–
	alternative methods	17	34.0	34.0	–
	methods when food was not tolerated	20	40.0	40.0	–
	food diary	24	48.0	48.0	–
	others	3	6.0	6.0	–
					
Occupation	not specified	1	2.0	2.0	2.0
	unemployed	2	4.0	4.0	6.0
	vocational training	4	8.0	8.0	14.0
	job	31	62.0	62.0	76.0
	homemaker	7	14.0	14.0	90.0
	retired	5	10.0	10.0	100.0
					
Use of the internet to obtain information on ingredients	yes, used	28	56.0	56.0	56.0
	no, not used	22	44.0	44.0	100.0

**Table 2.** Response rate for economic BELANA items in the pilot survey.

n = 50		Frequency	Percentage	Valid percentage	Accumulated percentage
ITEM 1: Specification of expenses for medical treatment	yes	47	94.0	100.0	100.0
	not specified	3	6.0		
					
ITEM 2: Specification of drug use	yes	50	100.0	100.0	100.0
	not specified	0	0.0		
					
ITEM 3: Specification of monthly extra costs for purchase special food	yes	42	84.0	100.0	100.0
	not specified	8	16.0		
					
ITEM 4: Specification of days with reduced activities of daily living	yes	38	76.0	100.0	100.0
	not specified	12	24.0		
					
ITEM 5: Specification of days on which they had to see a doctor	yes	47	94.0	100.0	100.0
	not specified	3	6.0		
					
ITEM 6: Specification of days they had to stay in a hospital	yes	50	100.0	100.0	100.0
	not specified	0	0.0		

**Table 3.** Selected economic parameters in the BELANA pilot study.

Item number	1	3	5	6
n = 50	Expenses for medical treatment within the past 4 months (EUR)	Monthly extra costs for food (EUR)	Days the patient had to see a doctor within the past 4 months	Days the patient had to stay in hospital within the past 4 months
Valid	47 (94%)	42 (84%)	47 (94%)	50 (100%)
Missing	3 (6%)	8 (16%)	3 (6%)	0 (0%)
Mean value	80.79	100.06	2.89	0.04
Mean standard deviation	16.42	15.32	0.67	0.04
Standard deviation	112.57	99.33	4.62	0.28
Variance	12672.04	9865.39	21.358	0.8
Minimum	0	0	0	0
Maximum	500	500	20	2
